# Altered interactions between attentional networks in older adults with mild cognitive impairment

**DOI:** 10.1016/j.heliyon.2024.e41329

**Published:** 2024-12-18

**Authors:** Ling Ma, Hao He, Yuehong Qiu, Yiqi Chen, Qing Guan

**Affiliations:** a School of Psychology, Shenzhen University, Shenzhen, China; b School of Education, South China Business College Guangdong University of Foreign Studies, Guangzhou, China; c Shenzhen-Hong Kong Institute of Brain Science-Shenzhen Fundamental Research Institutions, Shenzhen, China; d Department of Psychology, University of Mannheim, Mannheim, Germany; e College of Psychology, Liaoning Normal University, Dalian, China

**Keywords:** Mild cognitive impairment, Attentional networks, Alerting, Orienting, Executive control

## Abstract

Previous research using the Attention Network Test (ANT) paradigm has indicated that older adults with mild cognitive impairment (MCI) experience declines in attentional performance across the three core networks: alerting, orienting, and executive control, primarily focusing on main effects. The present study sought to expand these findings by exploring whether interactions between these networks are also affected in the presence of MCI. To achieve this, we used the Revised Attention Network Test (ANT-R) to examine both the individual attentional networks and their interactions in 21 older adults with MCI and 27 healthy controls (HCs) matched on demographic variables. Results indicated that the MCI group exhibited lower accuracy than the HC group in both executive control and orienting functions. In addition, the MCI group demonstrated a weakened interaction between the orienting and executive control networks, suggesting that valid cues are less effective in facilitating conflict resolution for individuals with MCI compared to HCs. No significant group differences were observed for the interaction between the executive control and alerting networks, or between the flanker conflict effect and the location conflict effect within the executive control network. This study extends previous findings by identifying changes related to MCI in the interaction between the orienting and executive control networks, highlighting the challenges individuals with MCI face in utilizing spatial cues during attentional processing.

## Highlights

• MCI-related changes in interactions between attentional networks were examined.
• Reduced orienting-executive control interaction was observed in the MCI group.
• Older adults with MCI have difficulty using spatial cues in conflict resolution.

## 1 Introduction

Mild cognitive impairment (MCI) represents an early stage of Alzheimer's disease (AD) [1]. Beyond the widely recognized symptom of memory loss, individuals with MCI often exhibit deficits in attentional functions [2,3]. Attention enables individuals to manage tasks quickly and accurately by prioritizing relevant information and ignoring irrelevant distractions [4], which is essential for daily functioning [5]. Posner and colleagues introduced the attention network theory, which conceptualizes attention as comprising three functionally interrelated but anatomically distinct networks: alerting, orienting, and executive control [4]. Within this theoretical framework, the Attention Network Test (ANT) was developed and has been widely used in both fundamental and clinical studies of neurodevelopmental disorders (e.g., attention deficit hyperactivity disorder [6]), neurodegenerative diseases (e.g., AD [7] and Parkinson's disease [8]), and psychiatric disorders (e.g., depression [9] and schizophrenia [10]).

Among the attentional networks, the alerting system is responsible for initiating and maintaining a heightened state of readiness in anticipation of an event, which is associated with norepinephrine activity within cortical areas involving thalamic, frontal, and parietal regions [4,11]. Following exposure to a warning cue, physiological changes, including shifts in heart rate and brain oscillations, induce a state of alertness that enhances response performance [12]. The orienting network is involved in directing attention toward specific stimuli, facilitating the processing of sensory inputs by spatially aligning attention to cues indicating target locations [13]. Orienting includes three key operations: disengaging from a current focus, shifting attention to a new location, and re-engaging attention on a new location or target [14]. These processes involve different brain regions, with the dorsal frontoparietal network playing a role in attentional shifts, while both ventral and dorsal attention networks are implicated in reorienting to novel stimuli [[15], [16], [17], [18], [19]]. The difference in reaction time (RT) and accuracy between valid and invalid cued targets is referred to as the validity effect [13]. The executive control network allocates goal-directed attention to task-relevant stimuli and responses, resolving conflicts arising from competing stimuli or distractions [20]. This network relies on the anterior cingulate cortex (ACC) and lateral prefrontal cortex and is influenced by dopamine [[21], [22], [23]].

Previous studies have demonstrated changes in the three attentional networks in older adults with MCI, focusing on main effects. MCI groups showed decreased alerting effects compared to healthy controls (HCs) [24,25], although some studies did not observe such deficits [2,26]. For the orienting network, some studies reported a decreased ability to disengage attention from invalidly cued locations and a reduced validity effect in MCI groups compared to HCs [27,28], while other findings reported no group differences [29,30]. Deficits in the executive control network have been more consistently observed in MCI populations compared to HCs [2,31,32]. These findings generally reflects a broader pattern that executive control is impacted earlier than orienting and alerting in the progression of neurodegenerative cognitive impairment [3].

However, less is known about whether interactions between these networks are affected in the presence of MCI. Studies on healthy younger adults have shown that attentional networks interact in specific ways [33,34]. The alerting network, while serving to prepare attention, can inhibit the executive control network to some extent, yielding a larger flanker effect when an alerting cue is previously presented. The orienting network can facilitate the executive network, with valid cues increasing and invalid or uninformative cues decreasing the ability to resolve conflicts. Given the previously demonstrated impairments in alerting and orienting functions in older adults with MCI, it is plausible to hypothesize that these two factors will have less impact on executive control in MCI individuals, resulting in reduced interaction effects.

This hypothesis is supported by previous studies. One study, which examined changes in the interaction between the auditory alerting and orienting networks related to healthy aging and amnestic MCI, found that older adults showed a stronger influence of alerting on orienting compared to younger adults; however, the MCI patients did not show a significant interaction between these networks [24]. However, the executive control function was not examined in that study. In another study, MCI patients with subcortical vascular damage (svMCI) showed diminished interactions between the orienting and executive control networks, and between the alerting and executive control networks, compared to MCI patients without subcortical vascular damage (nvMCI) and HCs. In contrast, no significant differences were observed in these interactions between nvMCI and HCs [27]. Despite these findings, research examining how MCI impacts interactions between attentional networks remains scarce.

To further examine whether and how interactions between attentional networks are altered in individuals with MCI, this study used the Revised Attention Network Test (ANT-R, which allows for the assessment of both individual networks and their interactions) to compare a group of older adults with MCI and a group of demographically matched HCs. We hypothesized that if the MCI group showed a deficit in alerting, the interaction between the alerting and executive control networks would be reduced. Similarly, if the MCI group showed a weakened orienting function, their orienting-executive control interaction would be decreased.

## 2 Material and methods

### 2.1 Participants

This study was a part of a long-term cognitive aging project conducted by our lab in the city of Shenzhen, China, from December 2022 to December 2024. Elderly residents from the community, aged 60 years and above, with at least five years of formal education (a prerequisite for neuropsychological assessments), voluntarily enrolled in this project. Participants underwent a community-based face-to-face interview to collect demographic information, medical records, and cognitive profiles. Cognitive profiles included general cognitive ability (assessed by a Chinese version of the Mini-Mental State Examination [MMSE]) [34], cognitive function across five domains (memory, attention, executive function, visuospatial ability, and language), subjective cognitive decline (self-reported and/or by informants), and activities of daily living (ADL, assessed by a combined version of the Physical Self-Maintenance Scale and the Instrumental Activities of Daily Living Scale, IADL) [35].

The assessment of cognitive function in this study covered five domains: memory, attention, executive function, language, and visuospatial ability. Memory was assessed using the Auditory Verbal Learning Test (AVLT) [36] and the Rey-Osterrieth Complex Figure Recall Test (ROCFT Recall) [37]. Attention was evaluated using the Trail Making Test Part A (TMT-A) [38] and the Symbol Digit Modalities Test (SDMT) [39]. Executive function was assessed using the Trail Making Test Part B (TMT-B) [38] and the Stroop Test (ST) [40]. Language abilities was examined using the Category Verbal Fluency Test (CVFT) [41] and the Boston Naming Test (BNT) [42]. Visuospatial skills were measured using the Rey-Osterrieth Complex Figure Copy Test (ROCFT Copy) [37] and the Clock Drawing Test (CDT) [43]. All assessments were conducted following the Chinese versions standardized by the Beijing Aging Brain Rejuvenation Initiative (BABRI) [44,45].

MCI was identified following Petersen's criteria [46], which include the following: (1) subjective cognitive complaints about cognitive difficulties; (2) overall cognitive performance within normal range (MMSE score of 24 or higher); (3) preserved ability to perform daily activities (scores of zero on both the ADL and the IADL); (4) evidence of cognitive deficits in at least one domain (scores on two tests in any domain falling more than 1.5 standard deviations (SD) below the norms adjusted for age and education in the Chinese elderly population) [46,47]; and (5) the absence of diagnosed neurodegenerative disease, psychiatric conditions, significant neurological disorders, or substance abuse [1]. To ensure that non-MCI patients with attentional or executive deficits did not influence the performance of control group, we excluded individuals from the control group who scored more than 1.5 SDs above the mean for reaction time (RT) or more than 1.5 SDs below the mean for count-based measures on any test of the attention and executive function domains.

A total of 21 older adults diagnosed with MCI (12 females, mean age = 65.34 ± 4.17 years, years of schooling = 9.38 ± 2.94 years) and 27 older adults with normal cognition (20 females, mean age = 64.81 ± 4.13 years, years of schooling = 10.07 ± 3.28 years, as the demographically matched HC group) were included in this experiment. Due to limited resources and the availability of older adults with MCI, we did not calculate the sample size corresponding to sufficient statistical power in advance. Instead, we heuristically estimated the range of sample sizes based on previous studies on aging and neurodegenerative cognitive decline on the ANT tasks [3,8,24,48]. The actual sample size in this study fell within the range of sample sizes observed in these studies: 32.32 ± 27.34 for healthy older adults and 22.92 ± 9.67 for participants with MCI. No significant differences in demographic variables were found between groups. All participants were reported to be right-handed and to have either normal or corrected-to-normal vision.

### 2.2 Ethical approval

The protocol was approved by the Medical Ethics Committee of Medical School, Shenzhen University (PN-202200120). Each participant provided written informed consent.

### 2.3 The Revised Attention Network Test (ANT-R)

**Fig. 1:**
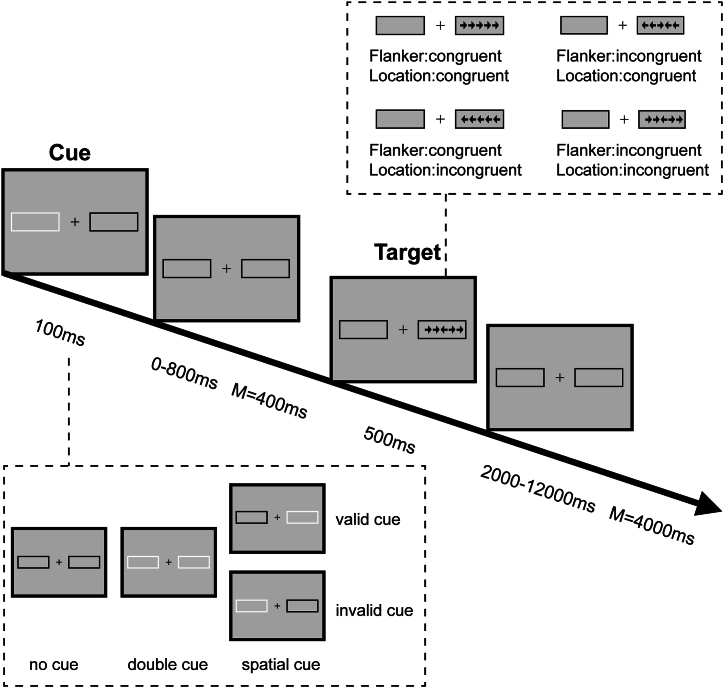
**Schematic of the Revised Attention Network Test (ANT-R).** Cue manipulations are shown in the bottom-left dashed rectangle. Flanker and location conflict manipulations are shown in the top-right rectangle. A schematic description of a trial in the ANT-R is shown in the central area. Participants are instructed to indicate the direction of the central arrow (target) (left or right).

We used the ANT-R program developed by Fan et al. [49], as illustrated in Fig. 1. Each trial began with a fixation cross displayed at the center of the screen, flanked by two rectangular boxes on the left and right. In cued trials, one or both boxes flashed for 100 ms, potentially facilitating target detection. In no-cue trials, neither box flashed. After a variable delay (0, 400, or 800 ms; mean delay = 400 ms), the target stimulus appeared in one of the rectangular boxes. The target stimulus consisted of a row of five horizontal black arrows displayed for 500 ms. The central arrow (target) was flanked by two arrows on each side (flankers), which either pointed in the same direction (congruent condition) or the opposite direction (incongruent condition). Participants were instructed to respond as accurately and quickly as possible by pressing an appropriate mouse button to indicate the direction (left or right) of the central arrow. Responses were recorded within a 1700 ms window following the onset of the target and flankers. The interval between the offset of the target and the start of the next trial was systematically varied, following an approximate exponential distribution with a mean of 4000 ms and a range of 2000 to 12,000 ms (10 intervals from 2000 to 4250 ms with 250 ms increments, plus a 4750 ms interval and a 12,000 ms interval). On average, each trial lasted 5000 ms.

The experiment comprised four blocks with a total of 288 trials distributed across four cue conditions. In the valid-cue condition (144 trials), the flashing box indicated the same location as the stimuli arrows, while in the invalid-cue condition (48 trials), the flashing box appeared in the opposite location. This probability-unbalanced design was intended to create a strong expectation of the target location and enhance the cost of attentional shifting, thus facilitating the examination of the orienting network. In the double-cue condition (48 trials), both boxes flashed, whereas in the no-cue condition (48 trials), neither box flashed. Within each block, cue types were counterbalanced to ensure equal probabilities of each cue type following any other cue type. Both RT and accuracy were recorded.

The test was administered on a PC using E-Prime™ software (Psychology Software Tools, Pittsburgh, PA). Participants began with a brief practice session, in which step-by-step instructions explaining the cue and target conditions were provided, followed by six practice trials with an unlimited response time window, and then 32 practice trials with the same timing parameters as the formal test. Practice continued until participants achieved a minimum accuracy of 80 %. Once this criterion was met, participants proceeded to the formal test.

### 2.4 Behavioral data analysis

Behavioral indicators were calculated using the MATLAB R2023a (The MathWorks, Inc., 2023). For each participant, the mean and SD of accuracy and RT were calculated for each condition. Trials with missing responses or RTs that deviated by more than ±3 SDs from the mean RT for a given condition were excluded from the analysis. The function of each attentional network was operationally defined as the difference in performance (in terms of RT and accuracy) between a given condition and its corresponding baseline condition. Table 1 provides an example of these calculations using the RT measure. The same approach was applied to the accuracy measure.

**Table 1:** The operational definitions of the three attentional networks and their interactions on the ANT-R (taking RT as an example).

Effects	Calculations
Alerting	RT _no-cue_ – RT _double-cue_
Orienting	RT _double-cue_ – RT _valid-cue_
Disengaging	RT _invalid-cue_ – RT _double-cue_
Validity	RT _invalid-cue_ – RT _valid-cue_
Flanker conflict	RT _flanker-incongruent_ – RT _flanker-congruent_
Location conflict	RT _location-incongruent_ – RT _location-congruent_
Alerting × flanker	(RT _no-cue, flanker-incongruent_ – RT _no-cue, flanker-congruent_) – (RT _double-cue, flanker-incongruent_ – RT _double-cue, flanker-congruent_)
Orienting × flanker	(RT _double-cue, flanker-incongruent_ – RT _double-cue, flanker-congruent_) – (RT _valid-cue, flanker-incongruent_ – RT _valid-cue, flanker-congruent_)
Disengaging × flanker	(RT _invalid-cue, flanker-incongruent_ – RT _invalid-cue, flanker-congruent_) – (RT _double-cue, flanker-incongruent_ – RT _double-cue, flanker-congruent_)
Validity × flanker	(RT _invalid-cue, flanker-incongruent_ – RT _invalid-cue, flanker-congruent_) – (RT _valid-cue, flanker-incongruent_ – RT _valid-cue, flanker-congruent_)
Flanker × location	(RT _flanker-incongruent; location-incongruent_ – RT _flanker-congruent; location-incongruent_) – (RT _flanker-incongruent; location-congruent_ – RT _flanker-congruent; location-congruent_)
Alerting × location	(RT _no-cue, location-incongruent_ – RT _no-cue, location-congruent_) – (RT _double-cue, location-incongruent_ – RT _double-cue, location-congruent_)
Orienting × location	(RT _double-cue, location-incongruent_ – RT _double-cue, location-congruent_) – (RT _valid-cue, location-incongruent_ – RT _valid-cue, location-congruent_)
Disengaging × location	(RT _invalid-cue, location-incongruent_ – RT _invalid-cue, location-congruent_) – (RT _double-cue, location-incongruent_ – RT _double-cue, location-congruent_)
Validity × location	(RT _invalid-cue, location-incongruent_ – RT _invalid-cue, location-congruent_) – (RT _valid-cue, location-incongruent_ – RT _valid-cue, location-congruent_)

Note: The definitions of effects on accuracy follow the same calculations.

The alerting network was quantified by calculating the difference between the no-cue and double-cue conditions (i.e., subtracting the latter from the former; same below). A larger effect size indicates stronger alerting function [49]. The orienting network was broken down into three components: “moving + engaging”, disengaging, and validity. The “Moving + engaging” component, representing the “orienting” effect or the benefit of shifting attention, was calculated as the difference between double-cue and valid-cue conditions. A larger effect size suggests a stronger facilitation of target response under the valid-cue condition. Disengaging, which represents the cost of disengaging attention from a current focus, was calculated as the difference between the invalid-cue and double-cue conditions. A higher value indicates a greater difficulty in shifting attention. The validity effect, which represents the combined cost of disengaging and shifting attention, was calculated as the difference between the invalid-cue and valid-cue conditions. A larger value typically indicates a greater cost associated with reorienting attention. For the executive control (conflict) network, the conflict effect was calculated as the difference between the incongruent and congruent conditions, including both flanker and location conflict effects. Smaller conflict effect sizes reflect better executive control over conflicting information.

Interactions between the conflict network and the alerting and orienting networks were also analyzed. The alerting × flanker conflict was calculated by subtracting the flanker effect in the double-cue condition from that in the no-cue condition. A positive value for RT or a negative value for accuracy indicates a positive effect of alerting on flanker conflict processing. The orienting × flanker conflict was measured by subtracting the flanker effect in the valid-cue condition from that in the double-cue condition. The validity × flanker conflict was calculated by subtracting the flanker effect in the valid-cue condition from that in the invalid-cue condition. The disengaging × flanker conflict was measured by subtracting the flanker effect in the double-cue condition from that in the invalid-cue condition. Positive values for RT or negative values for accuracy of the above three effects suggest enhanced spatial attentional function leading to more efficient conflict resolution. The operational definitions of the interactions with the location conflict effect were calculated in the same way as that with the flanker effect. The flanker × location was calculated as the difference in the flanker effect between the location-incongruent and location-congruent conditions. A positive RT value or a negative accuracy value indicates that the flanker conflict effect is reduced in the location-congruent condition compared to the location-incongruent condition.

Two-tailed paired-sample t-tests were performed to examine significant main effects of attentional networks and their interactions in each of the MCI and HC groups. To examine group differences in the attentional network main effects and interactions, we first checked the normality of distributions for all the accuracy and RT measures using the Shapiro-Wilk method, and then performed independent sample t-tests (with Levene's tests for equality of variances) for variables that met the assumption of normality and Mann-Whitney U tests for variables that did not meet the normality assumption. These tests were performed using IBM SPSS 25.0 Statistics. Effect sizes for t-tests were calculated using Cohen's d, while effect sizes for Mann-Whitney U tests were quantified using the rank-biserial correlation, calculated as $r=1-2U/\left(n_{1}\times n_{2}\right)$ [50,51]. The rank-biserial correlation ranges from 0 to 1, with a value of 1 representing a perfect association between the dichotomous variable (group) and the ranking variable (accuracy or RT).

We acknowledge that conducting the multiple tests increases the likelihood of type I errors (false positives). However, we chose not to apply corrections to our results for several reasons. First, although corrections may reduce the likelihood of type I errors, they may also reduce statistical power. Given that some of the effects and group differences observed in the ANT-R are relatively small, the use of such corrections could obscure potentially meaningful differences. We chose to balance the risks of both type I and type II errors to ensure that significant effects were not missed. Second, we reported effect sizes and Bayes factors in addition to p-values. This approach provides additional context for interpreting the magnitude and practical significance of our findings and helps to assess the robustness of the results independently of p-value adjustments. Technically, we used the bruceR package [52], which calculates Bayesian factors using the *ttestBF* function from the BayesFactor package [53]. Default parameters were used for these computations. Finally, we would compare our results with those of previous studies to validate our findings against the established literature.

## 3 Results

### 3.1 Performance on neuropsychological tests

The MCI group demonstrated significantly lower performance across all neuropsychological tests compared to the HC group (Table 2).

**Table 2:** Comparisons of performance on neuropsychological tests between the MCI group and the HC group.

	MCI (n = 21)	HC (n = 27)	t (χ^2^)	p	d (φ)	BF_10_
***Demographics***						
Gender (females/males)	12/9	20/7	0.86^a^	0.35	0.10	1.00^b^
Age (year)	65.34 (4.17)	64.81 (4.13)	−0.76	0.45	−0.13	0.31
Education (year)	9.38 (2.94)	10.07 (3.28)	0.43	0.66	0.22	0.37
***Cognitive Measures***						
Rey-Osterrieth Complex Figure Recall Test	7.52 (4.75)	16.56 (7.66)	5.01	<0.001	1.42	840
Auditory Verbal Learning Test	19.86 (8.37)	35.04 (10.29)	5.49	<0.001	1.62	2910
Rey-Osterrieth Complex Figure Copy Test	28.10 (7.12)	33.67 (2.56)	3.42	0.002	1.04	59.8
Clock Drawing Test	19.90 (5.06)	23.93 (4.23)	3.00	<0.001	0.86	9.34
Category Verbal Fluency Test	14.71 (4.47)	19.67 (3.43)	4.20	<0.001	1.24	276
Boston Naming Test	22.05 (3.81)	25.22 (2.15)	3.41	0.002	1.02	43.3
Symbol Digit Modalities Test	23.14 (5.07)	37.96 (7.57)	8.10	<0.001	2.30	9.34 × 10^6^
Trail Making Test Part A (ms)	75.81 (26.75)	48.56 (9.61)	−4.45	<0.001	−1.36	1430
Trail Making Test Part B (ms)	209 (48.43)	144 (36.38)	−5.27	<0.001	−1.52	4110
Stroop Test (ms)	99 (39.69)	76 (15.69)	−2.54	0.018	−0.76	5.95

Data are shown as mean (SD). MCI: Mild Cognitive Impairment; HC: Healthy Control. ^a^ Pearson's Chi-squared test with Yates' continuity correction was used. ^b^ The Bayesian factor was computed using the *contingencyTableBF* function from the BayesFactor package.

### 3.2 Overall performance on the ANT-R

There was no significant group difference in grand mean RT across conditions, t(46) = 0.63, p = 0.53, Cohen's d = 0.18, BF_10_ = 0.34, although grand mean RT was slightly longer in the MCI group (M = 795 ms, SD = 132 ms) than in the HC group (M = 774 ms, SD = 94 ms). There was also no significant group difference in grand mean accuracy across all conditions, z = −1.49, p = 0.14, r = 0.25, BF_10_ = 1.56, although grand mean accuracy was lower in the MCI group (M = 0.90, SD = 0.10) than in the HC group (M = 0.95, SD = 0.06).

### 3.3 Main effects of attentional networks

**Fig. 2:**
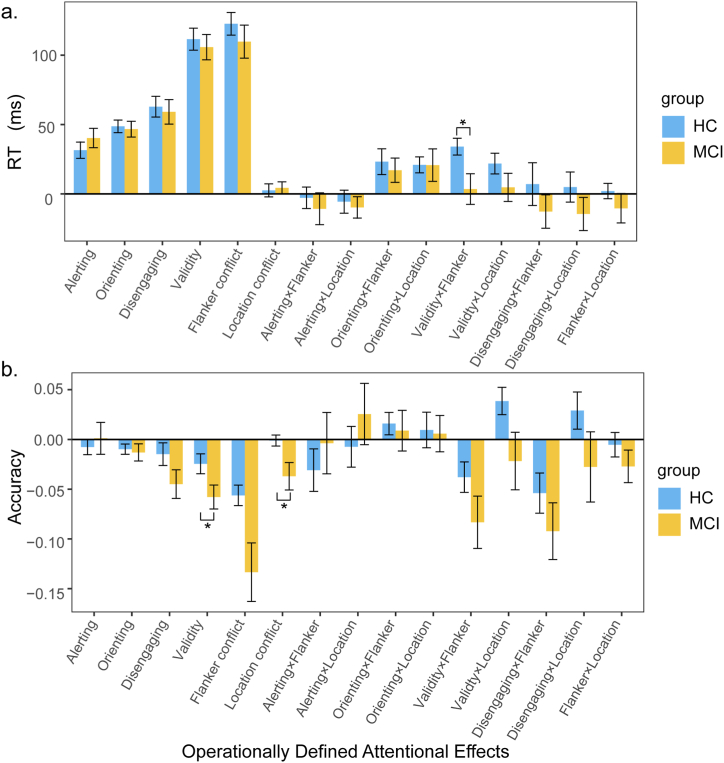
**A composite figure that illustrates the main effects and interactions of attentional networks on RT and accuracy in the MCI group and the HC group.** Error bars represent standard error. *∗*p *<* 0.05.

The main effects of the three attentional networks and their interactions on RT and accuracy for both groups are shown in Fig. 2a and b.

#### 3.3.1 The alerting network

The HC group showed a significant alerting effect on RT (M = 32 ms, SD = 31 ms)*,* t(26) = 5.37, p < 0.001, Cohen's d = 1.03, BF_10_ = 1720, but not on accuracy (M = −0.008, SD = 0.04), t(26) = −0.10, p = 0.33, Cohen's d = −0.20, BF_10_ = 0.32. For the MCI group, the alerting effect was also significant on RT (M = 40 ms, SD = 32 ms), t(20) = 5.80, p < 0.001, Cohen's d = 1.27, BF_10_ = 1980, but not on accuracy (M = 0.001, SD = 0.07), t(20) = 0.08, p = 0.94, Cohen's d = 0.02, BF_10_ = 0.23. There was no significant group difference in the alerting effect on either accuracy (z = −1.49, p = 0.14, *r* = 0.16, BF_10_ = 0.32) or RT (t(46) = −0.97, p = 0.34, Cohen's d = *-*0.29, BF_10_ = 0.42). The results suggest that there was no significant group difference in the alerting function which improved overall response speed rather than accuracy for both groups.

#### 3.3.2 The orienting network

*The orienting effect*. For the HC group, the orienting effect (moving + engaging) on RT (M = 49 ms, SD = 23 ms) was significant, t(26) = 10.77, p < 0.001, Cohen's d = 2.07, BF_10_ = 2.14 × 10^8^, whereas the effect on accuracy (M = −0.01, SD = 0.03) was not significant, t(26) = −1.89, p = 0.07, Cohen's d = −0.36, BF_10_ = 0.95. For the MCI group, the orienting effect on RT (M = 47 ms, SD = 26 ms) was significant, t(20) = 8.23, p < 0.001, Cohen's d = 1.79, BF_10_ = 1.79 × 10^5^, but the effect on accuracy (M = −0.01, SD = 0.04) was not significant, t(20) = −1.50, p = 0.15, Cohen's d = −0.33, BF_10_ = 0.60. There was no significant group difference in the effect size on either RT (t(46) = 0.29, p = 0.78, Cohen's d = 0.08, BF_10_ = 0.30) or accuracy (t(46) = 0.34, p = 0.74, Cohen's d = 0.10, BF_10_ = 0.30). These findings suggest that valid cues improved response speed, but not accuracy, compared to double cues in both groups. However, the effect size was not significantly different between the two groups.

*The disengaging effect*. The disengaging effect was significant on RT (M = 63 ms, SD = 39 ms, t(26) = 8.41, p < 0.001, Cohen's d = 1.62, BF_10_ = 1.85 × 10^6^) but not on accuracy (M = −0.01, SD = 0.06, t(26) = −1.30, p = 0.21, Cohen's d = −0.25, BF_10_ = 0.43) in the HC group. The MCI group showed significant disengaging effects on both RT (M = 59 ms, SD = 40 ms, t(20) = 6.70, p < 0.001, Cohen's d = 1.46, BF_10_ = 1.16 × 10^4^) and accuracy (M = −0.04, SD = 0.07, t(20) = −3.12, p = 0.005, Cohen's d = −0.68, BF_10_ = 8.44). There was no significant group difference in the effect size on RT (t(46) = 0.34, p = 0.75, Cohen's d = 0.09, BF_10_ = 0.32) or accuracy (z = −1.24, p = 0.22, r = 0.21, BF_10_ = 0.88). These results indicate that compared to double cues, invalid cues increased RTs for both groups, with no difference in the effect size between the groups. However, invalid cues decreased accuracy in the MCI group but they did not significantly affect HCs.

*The validity effect*. The HC group showed significant validity effects on both RT (M = 112 ms, SD = 41 ms, t(26) = 14.02, p < 0.001, Cohen's d = 2.70, BF_10_ = 5.62 × 10^10^) and accuracy (M = −0.02, SD = 0.05, t(26) = −2.44, p = 0.02, Cohen's d = −0.47, BF_10_ = 2.43). The MCI group also showed significant validity effects on both RT (M = 106 ms, SD = 42 ms, t(20) = 11.59, p < 0.001, Cohen's d = 2.53, BF_10_ = 3.94 × 10^7^) and accuracy (M = −0.06, SD = 0.06, t(20) = −4.79, p < 0.001, Cohen's d = −1.04, BF_10_ = 249). There was a significant group difference in the validity effect size on accuracy (t(46) = −2.17, p = 0.04, Cohen's d = −0.63, BF_10_ = 1.8) but not for RT (t(46) = 0.48, p = 0.64, Cohen's d = 0.14, BF_10_ = 0.30). Furthermore, in the valid-cue condition, there was no significant difference in accuracy between the MCI group (M = 0.91, SD = 0.10) and the HC group (M = 0.96, SD = 0.06), t(31.58) = 1.92, p = 0.06, Cohen's d = 0.57, BF_10_ = 1.5. In contrast, in the invalid-cue condition, accuracy was significantly lower in the MCI group (M = 0.85, SD = 0.14) than in the HC group (M = 0.93, SD = 0.09), t(32.73) = 2.29, p = 0.03, Cohen's d = 0.68, BF_10_ = 2.84. These results suggest that valid cues improved response speed and accuracy for both groups, while invalid cues had a greater negative effect on the accuracy for the MCI group compared to HCs.

#### 3.3.3 The executive control network

Significant flanker effects were found on both RT (M = 123 ms, SD = 42 ms, t(26) = 15.00, p < 0.001, Cohen's d = 2.89, BF_10_ = 2.54 × 10^11^) and accuracy (M = −0.06, SD = 0.05, t(26) = −5.46, p < 0.001, Cohen's d = −1.05, BF_10_ = 2150) in the HC group. Significant flanker effects were also found on RT (M = 110 ms, SD = 55 ms, t(20) = 9.21, p < 0.001, Cohen's d = 2.01, BF_10_ = 1.04 × 10^6^) and accuracy (M = −0.13, SD = 0.13, t(20) = −4.54, p < 0.001, Cohen's d = −0.99, BF_10_ = 149) in the MCI group. There was no significant group difference in either RT (t(46) = 0.93, p = 0.36, Cohen's d = 0.27, BF_10_ = 0.41) or accuracy (z = 1.64, p = 0.10, r = 0.28, BF_10_ = 5.23).

The location effect was not significant for either RT (M = 3 ms, SD = 24 ms, t(26) = 0.55, p = 0.59, Cohen's d = 0.11, BF_10_ = 0.23) or accuracy (M = −0.01, SD = 0.03, t(26) = −0.17, p = 0.86, Cohen's d = −0.03, BF_10_ = 0.21) in the HC group. In the MCI group, the location effect was not significant for RT (M = 4 ms, SD = 20 ms, t(20) = 0.99, p = 0.33, Cohen's d = 0.22, BF_10_ = 0.35), but it was significant for accuracy (M = −0.04, SD = 0.06, t(20) = −2.68, p = 0.01, Cohen's d = −0.59, BF_10_ = 3.75). The two groups did not differ significantly in the effect size on RT (z = −0.26, p = 0.80, r = 0.04, BF_10_ = 0.30), but they differed significantly in the effect size on accuracy (z = −2.14, p = 0.03, r = 0.36, BF_10_ = 4.41). Furthermore, there was no significant group difference in accuracy in the location congruent condition, t(46) = 1.49, p = 0.14, Cohen's d = 0.42, BF_10_ = 0.71, whereas in the location incongruent condition, the HC group showed significantly higher accuracy (M = 0.95, SD = 0.07) than the MCI group (M = 0.88, SD = 0.13), t(28.45) = 2.25, p = 0.03, Cohen's d = 0.67, BF_10_ = 2.88. These results suggest that the location conflict had a greater impact on the performance of participants with MCI than on that of HCs.

### 3.4 Interactions between attentional networks

#### 3.4.1 The alerting-conflict interactions

The alerting-flanker conflict interaction was not significant on either RT (M = −3 ms, SD = 40 ms, t(26) = −0.36, p = 0.72, Cohen's d = −0.07, BF_10_ = 0.22) or accuracy (M = −0.03, SD = 0.11, t(26) = −1.44, p = 0.16, Cohen's d = −0.28, BF_10_ = 0.50) in the HC group. For the MCI group, the alerting-flanker conflict interaction was also not significant on RT (M = −11 ms, SD = 53 ms, t(20) = −0.93, p = 0.36, Cohen's d = −0.20, BF_10_ = 0.33) or accuracy (M = −0.01, SD = 0.14, t(20) = −0.39, p = 0.70, Cohen's d = −0.09, BF_10_ = 0.24). There were no significant group differences in the effect size on RT (t(46) = 0.59, p = 0.56, Cohen's d = −0.17, BF_10_ = 0.33) or accuracy (t(46) = −0.75, p = 0.46, Cohen's d = 0.21, BF_10_ = 0.36).

The alerting-location conflict interaction was not significant for either RT (M = −6 ms, SD = 43 ms, t(26) = −0.67, p = 0.51, Cohen's d = −0.13, BF_10_ = 0.25) or accuracy (M = −0.01, SD = 0.02, t(26) = −0.36, p = 0.73, Cohen's d = −0.07, BF_10_ = 0.22) in the HC group. Also, the alerting-location conflict interaction was not significant on either RT (M = −10 ms, SD = 35 ms, t(20) = −1.25, p = 0.22, Cohen's d = −0.27, BF_10_ = 0.45) or accuracy (M = 0.03, SD = 0.14, t(20) = 0.83, p = 0.42, Cohen's d = 0.18, BF_10_ = 0.31) in the MCI group. There was no significant group difference in the interaction effect size for either RT (t(46) = 0.35, p = 0.73, Cohen's d = −0.10, BF_10_ = 0.30) or accuracy (z = −0.64, p = 0.53, r = 0.11, BF_10_ = 0.41). The results suggest that alerting did not improve the executive control network for either group.

#### 3.4.2 The orienting-, disengaging-, validity-conflict interactions

**Fig. 3:**
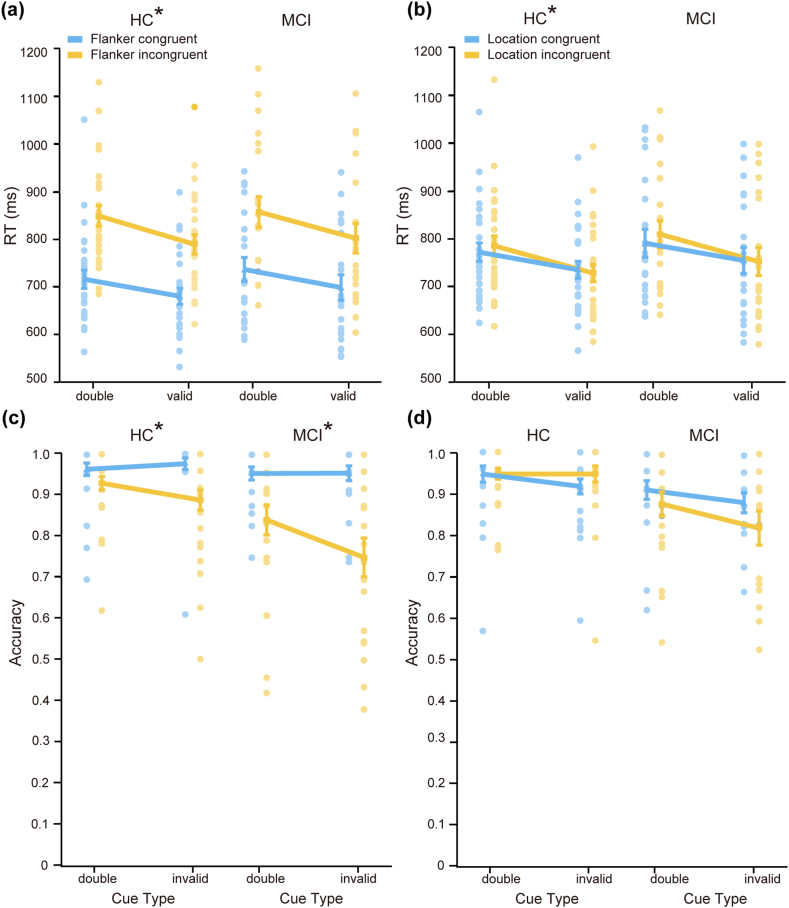
**A composite figure that illustrates the orienting-conflict and disengaging-conflict interactions in the MCI and HC groups.** (a) The orienting-flanker conflict interactions on RT. (b) The orienting-location conflict interactions on RT. (c) The disengaging-flanker conflict interactions on accuracy. (d) The disengaging-location conflict interactions on accuracy. The asterisks (∗) represent significant interactions. Each data point represents a single participant's performance in each condition, and error bars represent standard error.

*The orienting-conflict interaction.* For the HC group, the orienting-flanker conflict interaction was significant for RT (M = 23 ms, SD = 48 ms, t(26) = 2.50, p = 0.02, Cohen's d = 0.48, BF_10_ = 2.72; Fig. 3a), indicating that valid-cue resulted in a smaller flanker effect (107 ms) than double-cue (120 ms). For accuracy, however, the orienting-flanker conflict interaction was not significant (M = 0.02, SD = 0.06, t(26) = 1.42, p = 0.17, Cohen's d = 0.27, BF_10_ = 0.51). For the MCI group, the orienting-flanker conflict interaction was not significant for either RT (M = 17 ms, SD = 40 ms, t(20) = 1.95, p = 0.07, Cohen's d = 0.43, BF_10_ = 1.11; Fig. 3a) or accuracy (M = 0.01, SD = 0.09, t(20) = 0.44, p = 0.67, Cohen's d = 0.10, BF_10_ = 0.25). There was no significant group difference in the effect size on either RT (t(46) = 0.47, p = 0.64, Cohen's d = 0.14, BF_10_ = 0.32) or accuracy (t(46) = 0.32, p = 0.75, Cohen's d = 0.09, BF_10_ = 0.30).

For the HC group, the orienting-location conflict interaction on RT was significant (M = 21 ms, SD = 30 ms, t(26) = 3.64, p = 0.001, Cohen's d = 0.70, BF_10_ = 29.30; Fig. 3b), indicating that in the double-cue condition, RT was longer for incongruent locations than for congruent locations (location effect = 14 ms), whereas the pattern was reversed in the valid-cue condition (location effect = −7 ms). However, the orienting-location conflict interaction on accuracy was not significant (M = 0.01, SD = 0.09, t(26) = 0.54, p = 0.60, Cohen's d = 0.10, BF_10_ = 0.23) in the HC group. For the MCI group, the orienting-location conflict interaction was not significant on either RT (M = 21 ms, SD = 54 ms, t(20) = 1.77, p = 0.09, Cohen's d = 0.39, BF_10_ = 0.86; Fig. 3b) or accuracy (M = 0.01, SD = 0.08, t(20) = 0.32, p = 0.75, Cohen's d = 0.07, BF_10_ = 0.24). No significant group difference was found in the orienting-location conflict interaction on RT (t(46) = 0.02, p = 0.99, Cohen's d = 0.004, BF_10_ = 0.29) or accuracy (t(46) = 0.14, p = 0.89, Cohen's d = 0.04, BF_10_ = 0.29). The results suggest that orienting benefited executive control for the HC group but not for the MCI group, although no significant group difference was found in the effect size of the orienting-conflict interaction.

*The disengaging-conflict interaction.* For the HC group, the disengaging-flanker conflict interaction was not significant on RT (M = 11 ms, SD = 60 ms, t(26) = 0.94, p = 0.36, Cohen's d = 0.18, BF_10_ = 0.30), but was significant on accuracy (M = −0.05, SD = 0.11, t(26) = −2.66, p = 0.01, Cohen's d = −0.51, BF_10_ = 3.79; Fig. 3c) in the HC group, indicating that a smaller flanker effect was elicited in the double-cue condition (−0.03) than in the invalid-cue condition (−0.09). For the MCI group, the disengaging-flanker conflict interaction was not significant on RT (M = −14 ms, SD = 52 ms, t(20) = −1.20, p = 0.25, Cohen's d = −0.26, BF_10_ = 0.43) but was significant on accuracy (M = −0.09, SD = 0.13, t(20) = −3.24, p = 0.004, Cohen's d = −0.71, BF_10_ = 10.60; Fig. 3c), indicating that a smaller flanker effect was elicited in the double-cue condition (−0.11) than in the invalid-cue condition (−0.21). No significant group difference was found in the disengaging-flanker conflict interaction on RT (z = −1.29, p = 0.20, r = 0.22, BF_10_ = 0.42) or accuracy (z = −0.82, p = 0.41, r = 0.14, BF_10_ = 0.48).

The disengaging-location conflict interaction was not significant on either RT (M = 1 ms, SD = 48 ms, t(26) = 0.1, p = 0.92, Cohen's d = 0.02, BF_10_ = 0.21) or accuracy (M = 0.03, SD = 1.00, t(26) = 1.56, p = 0.13, Cohen's d = 0.30, BF_10_ = 0.58; Fig. 3d) in the HC group. For the MCI group, the disengaging-location conflict interaction was not significant on either RT (M = −16 ms, SD = 45 ms, t(20) = −1.63, p = 0.12, Cohen's d = −0.35, BF_10_ = 0.70) or accuracy (M = −0.03, SD = 0.16, t(20) = −0.78, p = 0.45, Cohen's d = −0.17, BF_10_ = 0.30; Fig. 3d). No significant group difference was found in the disengaging-location conflict interaction on either RT (t(46) = 1.20, p = 0.24, Cohen's d = −0.17, BF_10_ = 0.52) or accuracy (z = −1.38, p = 0.17, r = 0.23, BF_10_ = 0.72). The results showed that both groups had the reorienting cost on accuracy when dealing with the flanker conflict compared to the location conflict, but there was no significant group difference.

**Fig. 4:**
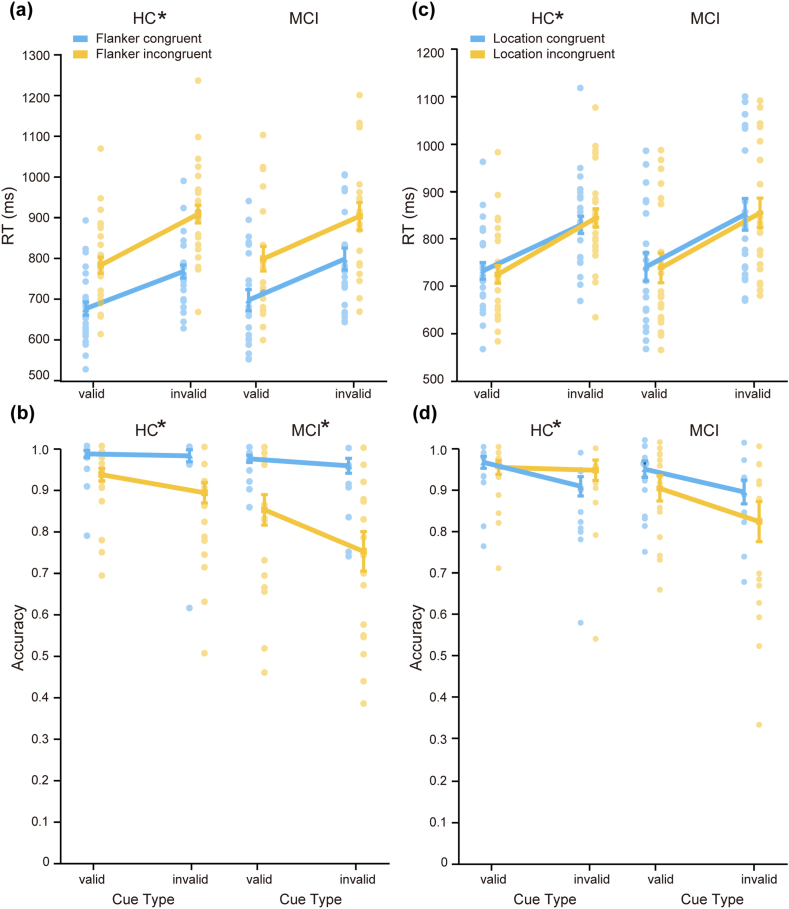
**A composite figure that illustrates the interactions between validity and executive control in the MCI and HC groups.** (a) The validity-flanker conflict interactions on RT. (b) The validity-location conflict interactions on RT. (c) The validity-flanker conflict interactions on accuracy. (d) The validity-location conflict interactions on accuracy. The asterisks (∗) represent significant interactions. Each data point represents a single participant's performance in each condition, and error bars represent standard error.

*The validity-flanker conflict interaction.* The validity-flanker conflict interactions were significant on both RT (M = 34 ms, SD = 32 ms, t(26) = 5.62, p < 0.001, Cohen's d = 1.08, BF_10_ = 3110; Fig. 4a) and accuracy (M = −0.04, SD = 0.08, t(26) = −2.48, p = 0.02, Cohen's d = −0.48, BF_10_ = 2.53; Fig. 4b) in the HC group, indicating that cue validity reduced the flanker effect on RT (141 ms vs. 107 ms for invalid-cue vs. valid-cue conditions) and accuracy (−0.09 vs. −0.05 ms for the invalid-cue vs. valid-cue conditions). For the MCI group, the validity-flanker conflict interaction was not significant on RT (M = 4 ms, SD = 51 ms, t(20) = 0.32, p = 0.76, Cohen's d = 0.07, BF_10_ = 0.24; Fig. 4a) but was significant on accuracy (M = −0.08, SD = 0.12, t(20) = −3.17, p = 0.005, Cohen's d = −0.69, BF_10_ = 9.65; Fig. 4c), indicating that cue validity reduced the flanker effect on accuracy (−0.21 vs. −0.12 for the invalid-cue vs. valid-cue conditions). A significant group difference in the validity-flanker conflict interaction was found for RT (t(31.66) = 2.43, p = 0.02, Cohen's d = 0.72, BF_10_ = 3.84) but not for accuracy (z = −1.48, p = 0.14, r = 0.25, BF_10_ = 0.78), indicating that the HC group showed a larger flanker effect for invalid cues than for valid cues but the MCI group showed a reduction in this effect. This result was predicted by our hypothesis that if older adults with MCI show reduced orienting function, their orienting-executive control interaction would be decreased.

*The validity-location conflict interaction.* The validity-location conflict interactions were significant on RT (M = 22 ms, SD = 39 ms, t(26) = 2.92, p = 0.007, Cohen's d = 0.56, BF_10_ = 6.16; Fig. 4c) and accuracy (M = 0.04, SD = 0.07, t(26) = 2.83, p = 0.009, Cohen's d = 0.54, BF_10_ = 5.37; Fig. 4d) in the HC group, indicating that in the invalid-cue condition, RTs were longer for incongruent locations than for congruent locations (location effect = 15 ms) and accuracy was higher for incongruent locations than for congruent locations (location effect = 0.03). However, this pattern was reversed in the valid-cue condition (the location effect on RT = −7 ms; the location effect on accuracy = 0). Whereas in the MCI group, the validity-location conflict interaction was not significant on either RT (M = 5 ms, SD = 7 ms, t(20) = 0.47, p = 0.65, Cohen's d = 0.10, BF_10_ = 0.25) or accuracy (M = −0.02, SD = 0.13, t(20) = −0.75, p = 0.46, Cohen's d = −0.16, BF_10_ = 0.30). No significant group differences were found in the effect size on RT (t(46) = 1.39, p = 0.17, Cohen's d = 0.40, BF_10_ = 0.63) or accuracy (z = −1.81, p = 0.07, r = 0.31, BF_10_ = 1.48).

The validity-conflict interaction results suggest that the MCI group showed a smaller validity effect compared to the HC group, who widely showed the advantage of valid cues over invalid cues for conflict processing across congruent/incongruent conditions and behavioral measures. This indicates that older adults with MCI use spatial cues less efficiently than HCs.

#### 3.4.3 The flanker-location conflict interaction

For the HC group, the flanker-location conflict interaction was not significant on either RT (M = 2 ms, SD = 23 ms, t(26) = 0.37, p = 0.71, Cohen's d = 0.07, BF_10_ = 0.22) or accuracy (M = −0.005, SD = 0.06, t(26) = −0.43, p = 0.67, Cohen's d = −0.08, BF_10_ = 0.23). For the MCI group, the flanker-location conflict interaction was not significant on either RT (M = −10 ms, SD = 48 ms, t(20) = −0.98, p = 0.34, Cohen's d = −0.22, BF_10_ = 0.05) or accuracy (M = −0.03, SD = 0.07, t(20) = −1.65, p = 0.11, Cohen's d = −0.36, BF_10_ = 0.69). There was no significant group difference in the flanker-location conflict interaction on either RT (z = −0.59, p = 0.55, r = 0.10, BF_10_ = 0.48) or accuracy (z = −1.19, p = 0.28, r = 0.19, BF_10_ = 0.47).

## 4 Discussion

Our results indicate that older adults with MCI retain normal alerting function, but have difficulty in disengaging from invalid cues, and their spatial processing accuracy is more susceptible to task-irrelevant information. Importantly, using the ANT-R, which includes the location factor absent in previous ANT versions, we confirm that older adults with MCI exhibit a reduction in the interaction between the orienting network and the executive control network (including both flanker and location conflict effects) compared to HCs. This change is associated with deficits in the orienting network, suggesting reduced efficiency in the use of spatial cues in older adults with MCI.

### 4.1 Group differences in main effects of attentional networks

Consistent with previous findings, our results indicated that the alerting network increased overall response speed without significant differences between the HC and MCI groups, suggesting that alertness is preserved in older adults with MCI [54]. This preservation might be due to neural compensation mechanisms, such as increased connectivity in the frontoparietal network [26]. For the orienting network, both groups showed that valid cues improved response speed while invalid cues decreased it, which did not differ between the groups. However, the MCI group showed a significant disengaging effect on accuracy (i.e., invalid cues decreased accuracy) while the HC group did not, leading to a significant difference in the validity effect between the groups. These results suggest that invalid cues have a stronger negative impact on accuracy in the MCI group, indicating difficulties in shifting attention from invalid cues to actual target locations [28,55].

In line with prior studies using the Simon task, which reported higher error rates in the MCI group compared to the HC group [56], our study also found reduced accuracy in the MCI group when resolving location conflicts. This finding suggests that deficits in attentional networks hinder the processing of location information. The deficits in the MCI group were mainly in accuracy, possibly due to impulsive decisions under time pressure, reflecting a lower response threshold associated with the reduced capacity of cognitive control [[57], [58], [59]].

### 4.2 The interaction between orienting and executive control

We focused on examining group differences in interactions between attentional networks, specifically the alerting-executive control interaction and the orienting-executive control interaction. Our findings revealed that the MCI group showed a reduction only in the orienting-executive control interaction. In general, valid cues minimize the need for disengaging and reorienting, thereby conserving attentional resources and enhancing executive control efficiency, represented by a reduced cognitive conflict effect for valid cues. Conversely, invalid cues increase the need for disengaging and reorienting, consuming attentional resources and decreasing executive control efficiency, resulting in an increased cognitive conflict effect.

This pattern was observed in the HC group for the interaction between orienting and executive control over conflict flankers. The HC group demonstrated normal orienting function as observed in younger adults [33], which facilitated the executive control network. Specifically, valid cues facilitated, and invalid cues distracted, the processing of flanker information, as shown by the orienting-flanker interaction on RT, the disengaging-flanker interaction on accuracy, and the validity-flanker interaction on both RT and accuracy. However, the MCI group showed deficits in the orienting network, with fewer significant interactions and a significantly attenuated validity-flanker interaction on RT compared to the HC group. This is consistent with previous studies in which significant interactions between validity and flanker congruency on RT were found for HC and MCI patients without subcortical vascular damage (nvMCI), but not for MCI patients with subcortical vascular damage (svMCI) [27]. These results suggest an inadequate use of cue information in older adults with MCI, leading to a decrease in the orienting-executive control interaction.

The MCI group also showed changes in the interaction between orienting and executive control over conflict locations. The location conflict effect in the ANT-R is essentially the Simon effect [60], where error rate and RT will increase when stimuli and responses differ in location compared to when they are at the same location [61]. Previous studies in younger adults showed that location effects were smaller for invalid cues than for valid cues [62], but the underlying mechanism has not been elucidated. In this study, the HC group showed the orienting-location interaction on RT and the validity-location interaction on both RT and accuracy, although the interactions were opposite to those previously found in younger adults (i.e., no Simon effects for valid cues). In contrast, no interaction was found between any of the three components of the orienting network and the location effect in the MCI group. The results showed that for participants with MCI, cue validity did not facilitate cognitive control during the Simon conflict processing, indicating ineffective use of cue information to enhance task performance.

Our behavioral results can be linked to previous findings from brain imaging studies to gain a deeper understanding of the neural underpinnings of deficits in the interaction between orienting and executive control. A previous fMRI study examining brain activation during ANT-R found that the orienting network was associated with the superior colliculus (SC) and frontal eye field (FEF), while the executive control network was associated with the frontoparietal network and cerebellum. Notably, the interaction between the two networks was associated with the pulvinar in the thalamus [63]. This indicates that the thalamus is crucial for coordinating information exchange and functional integration between these two networks. Moreover, previous MRI and pathological studies have been demonstrated neurodegenerative changes in key regions of the thalamus, including the anterodorsal, centromedial, and inferior pulvinar nuclei, the pregeniculate nucleus, and the lateral geniculate nucleus [[64], [65], [66], [67]]. These findings suggest that thalamus dysfunction may contribute to the impaired interaction between orienting and executive control in neurodegenerative cognitive impairment.

### 4.3 The interaction between alerting and executive control

We also tested whether older adults with MCI would show a reduction in the alerting-executive control interaction if they had a deficit in the alerting function. Our results showed that both groups exhibited the alerting effect, indicating that the alerting network functioned normally and that cues served as alerting signals for attention preparation. However, neither group showed a significant alerting-executive control interaction. This is consistent with a previous study using an auditory cue in a cognitive control task, which found no alerting-executive control interaction [68]. These results suggest that while cues increased general attentional preparation in older adults, they did not enhance deeper processes relevant to subsequent task attributes (e.g., detection of target locations). It is also possible that maintaining a constant state of alertness for simple tasks is effortful and demanding over time, leading to subjective tension or fatigue, according to the adaptive gain theory [69,70]. As a result, individuals may adaptively use phasic alertness to meet current task demands [70]. However, the optimal level of task-adapted alertness changes with age and cognitive conditions, implying that the level of alertness effective in young adults may not suffice for older adults. Future studies should investigate these possibilities further.

### 4.4 Limitation

Limitations of this study should be acknowledged. First, although we demonstrated MCI-related changes in attentional networks and their interactions, the sample size was relatively modest. Replication of our findings with larger sample sizes is essential for validation. Second, due to design constraints of the current version of the ANT-R, we were unable to examine the alerting-orienting interaction. Additionally, since previous studies have shown that the alerting effect elicited by auditory cues is greater than that elicited by visual cues, particularly in older adults [24,71,72] future studies should consider manipulating the alerting effect using different modalities. Third, contrary to previous findings in younger adults [33,61], we did not observe a significant flanker-location interaction on RT in either group in this study. As the mechanism underlying this interaction remains unclear and there is a lack of research on the flanker-location conflict interaction in both healthy aging and neurodegenerative cognitive impairment, future studies should explore this issue. Finally, the neural basis of MCI-related changes in attentional networks, which was beyond the scope of this study, remains an essential question to be addressed in future research.

## 5 Conclusion

Using the ANT-R, we thoroughly examined attentional network interactions as well as main effects in older adults with MCI compared to HCs. Our findings indicate that the MCI-related changes in attentional network interactions are primarily manifested in the interaction between the orienting network and the executive control network. This alteration results from a reduced efficacy in using spatial cues, including utilizing valid cues, disengaging attention from invalid cues, and inhibiting irrelevant information. In addition, weakened top-down control of conflict location information reflects the impairment in their spatial information processing.

## CRediT authorship contribution statement

**Ling Ma:** Writing – original draft, Visualization, Software, Methodology, Investigation, Formal analysis. **Hao He:** Writing – review & editing, Project administration, Methodology, Investigation, Funding acquisition, Formal analysis, Conceptualization. **Yuehong Qiu:** Writing – review & editing, Software, Investigation, Data curation. **Yiqi Chen:** Writing – review & editing, Methodology, Investigation, Data curation, Conceptualization. **Qing Guan:** Writing – review & editing, Supervision, Project administration, Funding acquisition, Conceptualization.

## Data availability statement

The data and code used in this study are available on the OSF (https://osf.io/7d5mb).

## Declaration of competing interest

The authors declare the following financial interests/personal relationships which may be considered as potential competing interests:Guan Qing reports financial support was provided by 10.13039/501100001809National Natural Science Foundation of China. He Hao reports financial support was provided by 10.13039/501100003453Guangdong Provincial Natural Science Foundation. Guan Qing reports financial support was provided by Ministry of Science and Technology of the People's Republic of China. Guan Qing reports financial support was provided by 10.13039/501100010877Shenzhen Science and Technology Innovation Commission. If there are other authors, they declare that they have no known competing financial interests or personal relationships that could have appeared to influence the work reported in this paper.
